# A clinical correlation between stature and posterior tooth length

**DOI:** 10.11604/pamj.2017.26.17.10436

**Published:** 2017-01-16

**Authors:** Smitha Reddy, Bhuvan Shome, Jayaprakash Patil, Pradeep Koppolu

**Affiliations:** 1 Department of Conservative and Endodontics, Sri Sai college of Dental Surgery, Vikarabad, Telangana, India; 2 Department of Preventive Dental Sciences, AlFarabi Colleges, Riyadh, KSA

**Keywords:** Stature, posterior tooth length, radioVisio graphy

## Abstract

**Introduction:**

Exploration and determination of the relationship between stature and length of tooth is essential in Paleontology, Forensic Odontology and Endodontology. This study aimed to determine any association between stature and posterior tooth length in a group of patients who required root canal treatment.

**Methods:**

Age, sex and standing height of adults were considered for posterior tooth length measurement. Molars and Premolars of apparently normal males (n=115 for molars, n= 75 for premolars) and females (n=124 for molars, n=80 for premolars), aged 20-50 years with intact cuspal morphology, which required RCT were selected for this study. Females and males were divided into 2 groups each based on their heights females > 155 cm and ≤ 155 cm, males > 165.10 and ≤ 165.10cm. The tooth length of permanent molars and premolars in both groups were measured using RVG and Electronic apex locator. Measurements obtained were compared separately for males and females using descriptive statistics and Pearson correlation coefficient.

**Results:**

In females MB, ML, D roots of molar showed significant association (P=0.021), (P=0.027), (P=0.010) and roots of premolars showed significant association (P=0.002), (P=0.006) between both the groups respectively In males MB, ML, D roots of molar showed significant association (P=0.009), (P=0.004), (P=0.015) and roots of premolars showed significant association (P=0.006), (P=0.020) between both the groups respectively.

**Conclusion:**

The present clinical study reveals that there is a positive association between stature and posterior tooth length in both males and females.

## Introduction

Investigating the relationship between tooth dimensions and body size is essential in forensic odontology and paleontology. “Large teeth necessitate large jaws, large jaws a large body” [1]. This statement though sounds logical, was not yet proved over the years. However, it is rational to speculate that taller people possess longer teeth, since the teeth contribute to the height of the face [2]. Although many studies compared tooth width with stature [3–6], not many reports are available on the relationship between stature and the posterior tooth length. Numerous studies indicated an existence of strong genetic influence on tooth dimensions [7, 8]. At the same time, environmental and dietary changes could also affect tooth morphology and dimensions [9, 10]. Living primate species to a large extent not only show distinctive differences in tooth form, but also show intra-species variation particularly between sexes [11]. While comparing fossil and contemporary hominoid dentitions, the possibility of a relationship between tooth size and body size must be considered. Several fossil hominoids have been thought to be giants because of their huge teeth [1, 12]. There are various reported correlations between parent’s height and children’s height; stature correlation to skull and jaw dimensions; length of various skeletal bones correlating to height or stature of a person; teeth and bones correlating to individual’s age and sex etc [13, 14]. Some researchers see the larger brain and tooth size as indicative of allometric changes due to increased body size. Though, these studies correlate various calcified and non- calcified structures of the human body, literature on the correlation between individual’s stature to the total length of the most calcified structure in human body that is the human tooth is still insufficient. There are various reports in literature comparing the stature of a person to the anterior teeth [15], but we could not find any article where the teeth with functional importance, the molars and premolars were investigated for a correlation between their posterior tooth lengths to the individual’s stature. This inspired us to explore the relationship between the posterior tooth length and body size in the modern man. The aim of this study is to investigate and determine the relationship between the height of a person and the posterior tooth length.

## Methods

**Figure 1:**
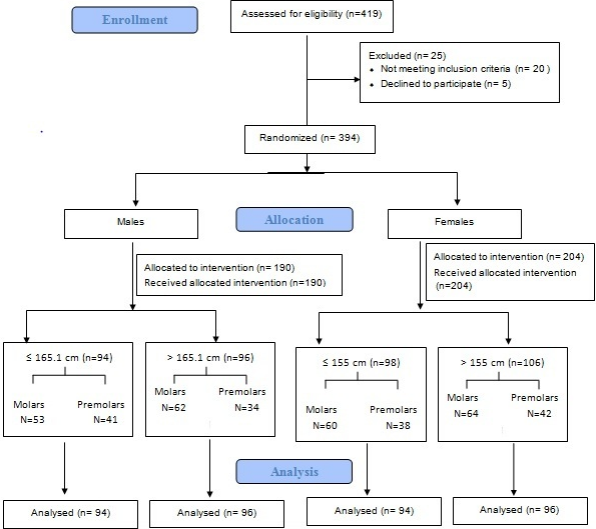
Flowchart

Molars and Premolars of apparently systemically healthy males (n=115 for molars, n =75 for premolars) and females (n=124 for mandibular first molars, n=80 for mandibular first premolars), aged 20-50 years with intact cuspal morphology, which required root canal therapy were selected for this study after getting the ethical clearance from the institute Ref. No. 311/SSCDS/IRB-E/OS 2012. Female and male groups were divided into 2 groups (Figure 1) each based on the average height of the individuals. Patients less than average height are classified as short, patients with height greater than average height are classified as tall [16, 17]. Females > 155 cm (Tall) and ≤ 155 cm (Short), males > 165.1 cm (Tall) and ≤ 165.1 cm (Short) [16, 17]. Teeth with attrition, root resorption, and severe root or canal curvatures or anomalies were excluded. Informed consents were obtained from the subjects selected prior to the study. Root Canal therapy was initiated and tooth length in millimetre (mm) was measured using Mesio-Lingual Cusp tip as an occlusal reference point for mandibular first molars whereas buccal cusp tips of mandibular first premolars were considered as the occlusal reference points. The working lengths of all the teeth were measured with long cone paralleling technique using RadioVisioGraphy (RVG) (Suniray) and which was further reconfirmed Electronic Apex Locator [Root ZX, J Morita] in mm. Where the instrument did not reach the anatomic apex in the RVG suggesting a curved root (buccal or lingual curvature), the apex was confirmed with the Electronic apex locator. Where the readings of Apex locator were shorter than the length measured on RVG image, it was considered as a lateral exit canal and the subjects were excluded from the study. The posterior tooth length was calculated by adding 1mm to the measured working length readings obtained with the Electronic apex locator [18]. Stature of the individual was measured as the vertical distance from vertex to the floor using stadiometer, a centimetre measuring tape with wall stop. Measurement was taken by making the subject stand upright on the floor barefooted, with the feet grounded flat on the floor and the head oriented in Frankfurt’s plane, while the shoulder and buttocks touching the wall behind. The movable gauze of the stadiometer mounted high on the wall was pulled downwards to contact the vertex in the mid sagittal plane [19, 20]. Hair was clipped to expose the scalp on the vertex in patients with dense hair. The working lengths were calculated for Mesiobuccal [MB], Mesiolingual [ML] and Distal [D] canal for mandibular first molars for males and females respectively. The length of roots of mandibular first Premolar was considered for males and females. Mean and standard deviation are calculated for all the parameters Comparisons were made between measurements recorded with respect to the gender using descriptive statistics, student’s t test and Relationships between the parameters were assessed by Pearson’s correlation coefficient. Information obtained was statistical analysed using IBM Statistical Program for Social Sciences Version 17.0 (SPSS Inc, Chicago Illinois, USA). All levels of statistical significance were set at p < 0.05.

## Results

**Figure 2:**
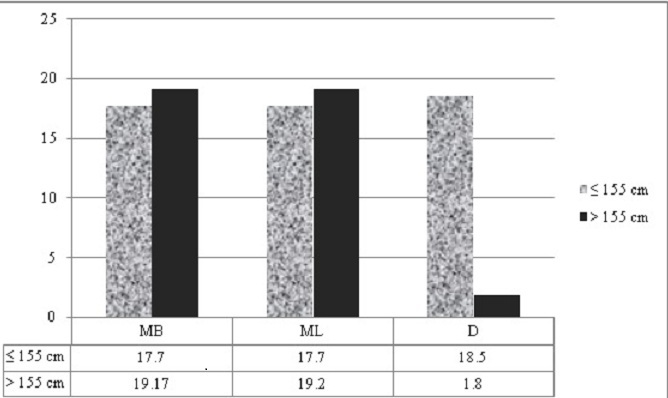
Length of canals in molars in female patients

**Figure 3:**
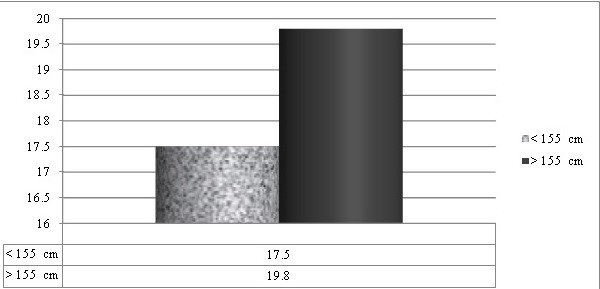
Length of canals in pre molars in female patients

**Figure 4:**
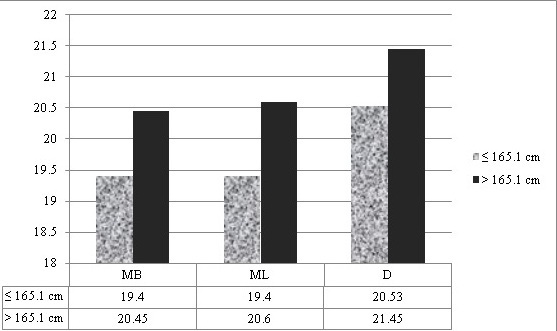
Length of canals in molars in male patients

**Figure 5:**
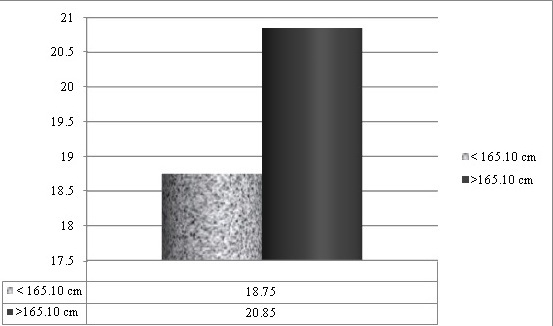
Length of canals in pre molars in male patients

The study sample included Molars and Premolars of apparently systemically healthy males and females aged 20-50 years, with intact cuspal morphology, which required root canal therapy were selected for this study (n=115 for mandibular first molars, n =75 for mandibular first premolars) and females (n=124 for mandibular first molars, n=80 for mandibular first premolars). Basic statistics of all variables showed that data were in normal distribution. The mean values of age and height of males were 41.4 year ± 2.24 and 169.278 ± 1.04 cm respectively, and those of females were 34.2 ± 3.44 years and 158.310 ± 1.98 cm respectively. Root length in molars showed significant association in female patients of both height groups (≤ 155 cm and > 155cm) Mean MB root length in molars showed significant association (P =0.021) in female patients of both height groups, Mean ML root length in molars showed significant association (P =0.027) in female patients of both height groups Mean D root length in molars showed significant association (P = 0.010) in female patients of both height groups (Table 1, Figure 2). Mean root length in mandibular first premolars showed significant association (P=0.002) in female patients of both height groups (Table 2, Figure 3). Mean MB root length in molars showed significant association (P = 0.009) in male patients of both height groups (≤ 165.10 cm and > 165.10cm), mean ML root length in molars showed significant association (P=0.004) in male patients of both height groups, mean D root length in molars showed significant association (P=0.015) in male patients of both height groups (Table 3, Figure 4). Mean root length in mandibular first premolars showed significant association (0.006) in male patients of both height groups (Table 4, Figure 5).

**Table 1:** Mean length of canals in molars (in mm) in female patients (n=124)

ROOT TYPE	≤ 155 cm
	MEAN ± SD
MB	17.7 ± 0.42
ML	17.7 ± 0.43
D	18.5 ± 0.49

* P = <0.05 statistically significant

**Table 2:** Mean length of canals in pre molars (in mm) in female (n=80)

≤ 155 cm	> 155 cm	P VALUE
MEAN±SD	MEAN±SD	
17.5 ± 0.72	19.80 ± 1.3	0.002^*^

* P = <0.05 statistically significant

**Table 3:** Mean length of canals in molars (in mm) in male patients (n=115)

ROOT TYPE	≤ 165.10 cm	> 165.10 cm	P VALUE
	MEAN±SD	MEAN±SD	
MB	19.4 ± 1.59	20.45 ± 1.11	0.009^*^
ML	19.4 ± 1.62	20.6 ± 1.09	0.004^*^
D	20.53 ± 1.36	21.45 ± 1.25	0.015^*^

* P = <0.05 statistically significant

**Table 4:** Mean length of canals in pre molars (in mm) in male patients (n=75)

≤165.10 cm	>165.10 cm	P VALUE
MEAN±SD	MEAN±SD	
18.75 ± 1.5	20.85 ± 1.76	0.006^*^

* P = <0.05 statistically significant

## Discussion

Investigating the correlation between tooth dimensions and body size is imperative in paleontology and forensic odontology. Do large animals have large teeth? In general they do, but how closely are big teeth correlated with body size? Garn and Lewis showed that for humans, tooth size and stature were only slightly correlated [5]. On the other hand, Bjork found a significant correlation between the total sum of maxillary tooth breadths and tibia length for the same species [21]. Anderson and Thompson suggested that tooth form did correlate with skeletal maturation in males and females. There was a significant correlation between late cessation of growth in body height and the absence of fifth cusp of the first mandibular molar. In the females, the absence of this cusp also was related to small canine width [22]. Garn *et al* found that percentage dimorphism is greatest for canine teeth, next greatest for molars and least for the lower incisors. To some extent, of course, these apparent differences in percentage sexual dimorphism may be attributable to such things as sampling error; differences in techniques of tooth measurement and degree of wear [23]. Initially X linked genes were thought to regulate tooth size and tooth form in humans and mice [24, 25]. Alvesalo found that the sex chromosomes have modifying effects not only on tooth shape, structure, and root size, but also influenced craniofacial form, body size and shape [24]. Later on he found that Y chromosome promotes both dentin and enamel growth, whereas effect of X chromosome seems to be limited to enamel. These differential effects of X and Y chromosomes on cell function and proliferation, especially that of Y chromosome on cell proliferation may be related to sexual dimorphism observed in tooth number, average crown and root size, crown morphology, and assuming genetic pleiotrophy, other somatic features such as stature growth and sex ratio at birth [26]. In the studies conducted by Szerep, there were evidences suggesting pituitary dwarfs exhibiting smaller teeth than the normal population in 10 of 13 comparisons [27]. Shortness of roots was also observed in a case reported by Seckel in 1960 in a patient with primordial short stature [28].

In the present study, the posterior tooth length was measured with long cone paralleling technique using RVG which was further reconfirmed by Electronic Apex Locator (Root ZX). Even though the most popular method in the literature has been the use of radiograph, with the advent of electronic method of apex locator, which are more reliable, combination of both methods were used to minimise error factors. It is accepted that apical constriction is on an average located 0.5-1.0mm short of radiographic apex [29, 30]. Since apex locators indicate the narrowest constriction; 1.0mm was added to all the readings to determine the anatomic tooth length. Stadiometer was used to estimate the stature of individuals, which is one of the most commonly used instrument for height measurement among various reported anthropometric studies [19, 20]. Teeth and bones provide clues to a person’s medical history. In general, anterior teeth fall off more frequently than the multirooted molars, which make the correlation of stature to multirooted functional teeth more important in forensic odontology. A positive association between individual’s stature and teeth could be very helpful to any dental surgeon in predicting the working length during root canal treatment, especially in difficult situations. The results of this study suggest believing on a hypothesis that, taller individuals shall possess longer teeth and vice versa. Though the results are very encouraging, further research is warranted. One of the possible limitations of the study would be not considering the calcifications in the root canal (if any) and the ledges which would have been formed due to the previous history of the treatment.

## Conclusion

The present clinical study reveals that there is a positive correlation between stature and posterior tooth length in both males and females. Further research and study with a larger sample is necessary in this regard. It is one of few studies which correlated the relation between height of the individual to the length of the tooth.

### What is known about this topic

- There are various reports in literature comparing the stature of a person to the anterior teeth, but there are no studies correlating between the posterior tooth lengths with functional importance like molars and premolars to the individual’s stature.

### What this study adds

- This study is the first of its kind to investigate and determine the relationship between the height of a person and the posterior tooth length;
- Results of this study can be used in investigating the forensics.
